# The impact of decreased expression of SVEP1 on abnormal neovascularization and poor prognosis in patients with intrahepatic cholangiocarcinoma

**DOI:** 10.3389/fgene.2022.1127753

**Published:** 2023-01-09

**Authors:** Liwei Chen, Yuchao He, Zhiqiang Han, Wenchen Gong, Xiangdong Tian, Lin Guo, Hua Guo, Tianqiang Song, Lu Chen

**Affiliations:** ^1^ Department of Hepatobiliary Cancer, Liver Cancer Research Center, Tianjin Medical University Cancer Institute and Hospital, National Clinical Research Center for Cancer, Key Laboratory of Cancer Prevention and Therapy, Tianjin’s Clinical Research Center for Cancer, Tianjin, China; ^2^ Department of Tumor Cell Biology, Tianjin Medical University Cancer Institute and Hospital, National Clinical Research Center for Cancer, Key Laboratory of Cancer Prevention and Therapy, Tianjin’s Clinical Research Center for Cancer, Tianjin, China; ^3^ Department of Anesthesiology, Tianjin Medical University Cancer Institute and Hospital, National Clinical Research Center for Cancer, Key Laboratory of Cancer Prevention and Therapy, Tianjin’s Clinical Research Center for Cancer, Tianjin, China; ^4^ Department of Pathology, Tianjin Medical University Cancer Institute and Hospital, National Clinical Research Center for Cancer, Key Laboratory of Cancer Prevention and Therapy, Tianjin’s Clinical Research Center for Cancer, Tianjin, China; ^5^ Department of Endoscopy, Tianjin Medical University Cancer Institute and Hospital, National Clinical Research Center for Cancer, Key Laboratory of Cancer Prevention and Therapy, Tianjin’s Clinical Research Center for Cancer, Tianjin, China; ^6^ Department of Genetics, School of Basic Medical Sciences, Tianjin Medical University, Tianjin, China

**Keywords:** intrahepatic cholangiocarcinoma, abnormal neovascularization, SVEP1, proliferation, prognosis

## Abstract

**Introduction:** Intrahepatic cholangiocarcinoma (ICC) is one of the most highly heterogeneous malignant solid tumors; it is generally insensitive to clinical treatment and has a poor prognosis. Evidence suggests that abnormal neovascularization in the tumor microenvironment is an important cause of treatment resistance as well as recurrence and metastasis, but the key regulatory molecules are still largely unknown and should be identified.

**Method:** We assessed the novel extracellular matrix protein (ECM) Sushi, von Willebrand factor type A, EGF and pentraxin containing 1 (SVEP1) expression pattern in the ICC by using immunohistochemistry. Multiplex immunofluorescence and Kaplan-Meier analysis were applied to explore the correlation between the low expression of SVEP1 and abnormal blood vessels and the clinical prognosis of ICC.

**Results:** Our study showed that the expression of SVEP1 in most ICC samples was relatively lower than in the adjacent tissues. Statistical analysis suggested that patients with decreased SVEP1 expression always had shorter overall survival (OS) and disease-free survival (DFS). Moreover, the expression of SVEP1 was negatively correlated with the proportion of abnormal neovascularization in the tumor microenvironment of the ICC. Consistently, the key molecule of promoting vascular normalization, Ang-1, is positively correlated with the SVEP1 expression and prognosis in the ICC. In addition, the proportion of high Ki-67 expression was higher in the ICC samples with low SVEP1 expression, suggesting that the SVEP1 low expressed sample is in a malignant phenotype with high proliferation.

**Conclusion:** This study reveals that SVEP1 is a promising prognostic biomarker for ICC and provides fresh insight into the role and potential new mechanism of abnormal neovascularization in ICC progression.

## 1 Introduction

Cholangiocarcinoma (CCA) is the sixth largest cancer-related death disease, accounting for approximately 2% of global cancer-related deaths yearly. In recent years, its incidence rate has increased rapidly worldwide, and only 30%–40% of patients in clinical practice can be removed by radical surgery at the time of initial diagnosis (Banales et al., 2020; Sasaki et al., 2021). According to different anatomical locations, cholangiocarcinoma is generally divided into ICC and extrahepatic cholangiocarcinoma (ECC). ICC is also the second common type of primary liver cancer, accounting for 10%–15% of all primary liver cancer (Sung et al., 2021). Although radical surgical resection is still the most important treatment method for cholangiocarcinoma at present, local recurrence and distant metastasis as well as the lack of effective comprehensive treatment strategies lead to poor long-term efficacy of ICC surgery. Nearly 50% of patients with cholangiocarcinoma experience recurrence within 1 year after surgery, and the 5 year survival rate is less than 5% (Nakamura et al., 2015).

The most recent studies indicate that in addition to the malignant biological characteristics of tumor cells, the interaction between the tumor cells and tumor microenvironment jointly led to the malignant progression of cancer (Li et al., 2020). Among them, angiogenesis in the microenvironment is an important feature of malignant tumors and also the key to maintaining tumor growth (Bejarano et al., 2021). The imbalance between promoting angiogenesis and anti-angiogenesis signals during tumor genesis and development usually leads to structural abnormalities of tumor neovascularization, which is characterized by disorder, immaturity, and leakage of blood vessels. The tumor microenvironment composed of abnormal neovascularization is conducive to tumor migration and therapeutic resistance and promotes tumor malignant progress (Sung et al., 2019; Kabir et al., 2021).

ECMs play an important role in tumor cell survival, tumor stem cell self-renewal, tumor angiogenesis, as well as tumor invasion and metastasis by regulating and changing cell adhesion, the composition of the extracellular matrix, and the tumor tissue microenvironment (Chen et al., 2019; Mohan et al., 2020). SVEP1 is a newly reported secreted multidomain ECM, which is involved in embryonic development, cell adhesion and differentiation, and maintaining tissue microenvironment homeostasis (Glait-Santar and Benayahu, 2012; Morooka et al., 2017; Samuelov et al., 2017). More importantly, in our previous studies, we found and reported that SVEP1 also plays a critical role in tumor heterogeneity (Guo et al., 2022), proliferation and metastasis (Chen et al., 2020), and tumor stem cell-like phenotype maintenance (Gong et al., 2022a). In this study, we firstly present the expression profile of SVEP1 and identified abnormal neovascularization in ICC. Then, we further explore the correlation between SVEP1 and abnormal neovascularization and its significance in predicting prognosis.

## 2 Materials and methods

### 2.1 Patients and specimens

The clinical ICC and paired normal samples were resected from 47 diagnosed patients with complete clinical information obtained from the Tianjin Medical University Cancer Institute and Hospital (Tianjin, China). This project was approved by the ethical committee of the Tianjin Medical University Cancer Institute and Hospital (No. bc2021290). Informed consent was obtained from all patients.

### 2.2 Multiplex immunofluorescence and analysis

The ICC paraffin samples were stained according to the protocol of PANO 6-plex IHC kit, cat#10236100100 (Panovue, Beijing, China). Primary antibodies were used in the following order: CD31 (Abcam, 281583, PPD520); α-SMA (Abcam, 124964, PPD570). Subsequently, sections were stained with DAPI, and slides were mounted and scanned by a PanoVIEW VS.200 slide scanner (Panovue, Beijing, China) with Olympus 40 × lens. Five to 10 representative multispectral images were selected and analyzed using inForm Advanced Image Analysis software (inForm 2.5.0; Akoya Biosciences, United States).

### 2.3 Immunohistochemistry

Immunohistochemistry (IHC) was performed as previously described (Chen et al., 2020). The IHC staining was used to detect the expression level of SVEP1, Ang-1, and Ki-67 in the paraffin samples from the ICC and paired normal tissues. The primary antibody included rabbit anti-SVEP1 polyclonal antibody (Abcam, cat#ab121677), rabbit anti-Angiopoietin-1 (Ang-1) polyclonal antibody (Bioss, cat#bs-0800R), and mouse anti-Ki-67 monoclonal antibody (Zhongshan Goldbridge Biotechno-logy CO., Ltd., Beijing, China). According to the signal distribution and intensity, the expression levels of SVEP1, Ang-1, and Ki-67 were scored by two pathologists using a blinded method. Moreover, the IHC score was used to analyze the relationship between SVEP1/Ang-1 and the overall survival time (OS) and disease-free survival (DFS) of the ICC patients. The IHC scoring rules of SVEP1 and Ang-1 are as follows: IHC staining intensity was evaluated in four classes (0 = none, 1 = weak, 2 = medium, and 3 = strong), and the percentage of positive cells was classified into four scales (0 = none, 1 < 30%; 2 = 30%–60%, and 3 = 60%–100%). The final IHC score was the product of the IHC staining intensity score and the percentage score. The group with the final IHC score >6 was defined as the high expression group, whereas the group with the final score ≤6 was defined as the low expression group. Ki-67 staining was located in the nucleus, and the percentage of Ki-67 positive cells was classified into four scales (0 = none, 1 < 15%; 2 = 15%—50%, and 3 = 50%—100%). The cutoff value for discriminating Ki-67^high^ and Ki-67^low^ was 15%.

### 2.4 Western blot assay

Western blotting was conducted as described previously (Chen et al., 2017; He et al., 2020).The following antibodies were used: anti-SVEP1 (1:500) from R&D (MAB97741), anti-GAPDH (1:1000) from Santa Cruz Biotechnology.

### 2.5 Gene set enrichment analysis

GSEA was performed to determine whether the SVEP1 mRNA level is related to tumor cell proliferation phenotype, on the basis of GSE132305 data sets for ICC using GSEA 4.1.0 (The Broad Institute of MIT and Harvard).

### 2.6 Statistical analysis

All data are shown as the means ± SDs. SPSS 26.0 (SPSS Inc., Chicago, IL) was used to evaluate the data. Survival curves were plotted using the Kaplan-Meier method and analyzed with the log-rank test. Differences between groups were evaluated by a two-tailed Student’s t-test or ANOVA. *p* < .05 was considered to indicate a statistically significant difference.

## 3 Results

### 3.1 Expression pattern of SVEP1 in ICC and its correlation with prognosis

**FIGURE 1:**
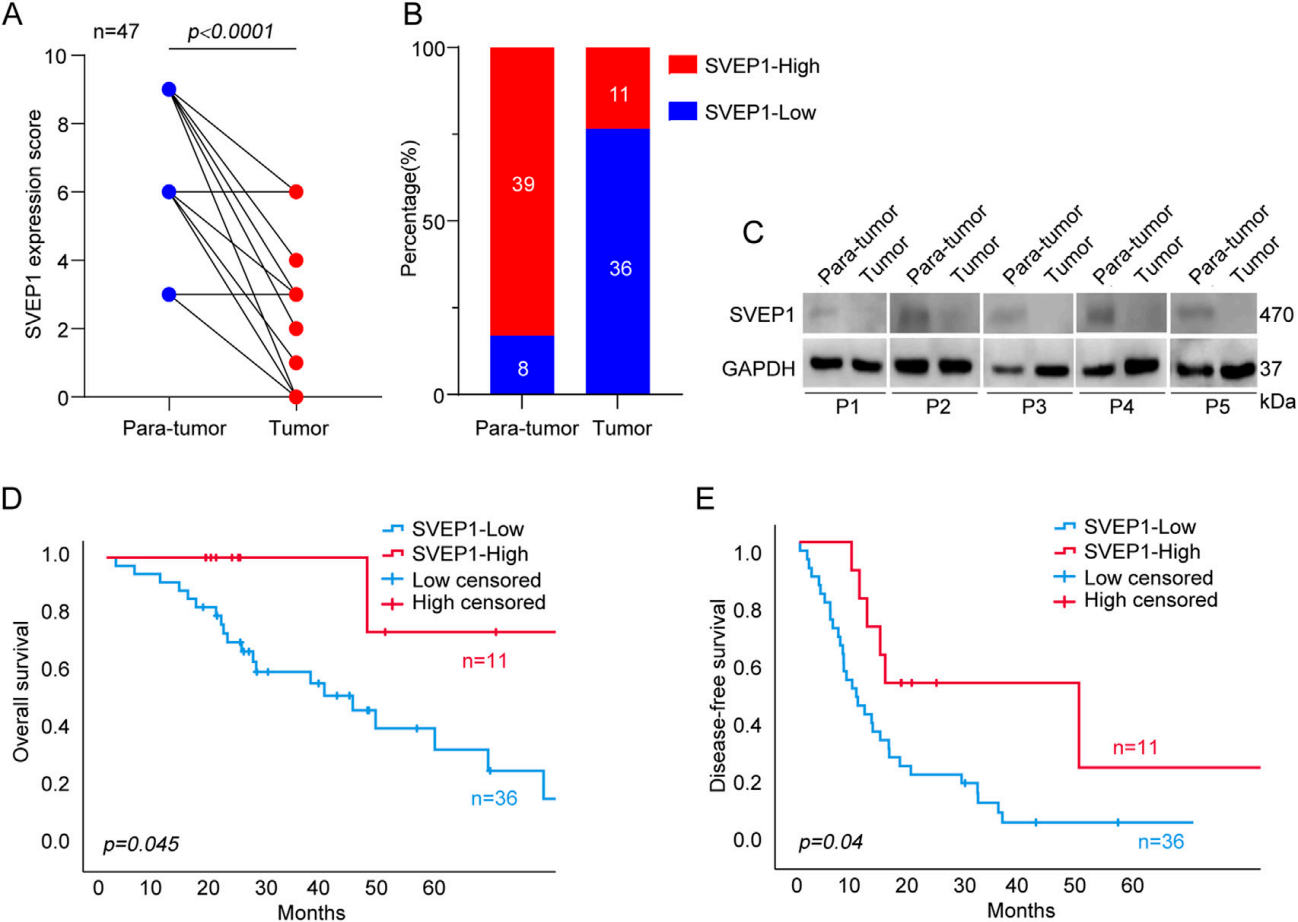
The expression pattern of SVEP1 in ICC and its correlation with prognosis. **(A)** The SVEP1 expression level based on the IHC score in tumor tissues compared with paired adjacent normal tissues. **(B)** The proportion of high and low expression of SVEP1 in tumor tissues compared with paired adjacent normal tissues. **(C)** The level of SVEP1 protein among five pairs of para-tumor and tumor by western blot assay. **(D–E)** Kaplan-Meier analysis comparing OS **(D)** and DFS **(E)** in the ICC patients with high and low expression of SVEP1.

We performed immunohistochemical (IHC) staining on 47 ICC tissues and paired adjacent normal tissues to determine the expression pattern of SVEP1 in the ICC. The result showed that SVEP1 was mainly expressed in the cytoplasm and extracellular matrix. Moreover, the expression level of SVEP1 decreased significantly in tumor tissues compared with paired adjacent normal tissues (Figure 1A, *p* < .0001). A total of 36 out of 47 (76.6%) ICC tissues exhibited SVEP1 low or negative expression. However, this proportion was substantially low in the adjacent normal tissues, only 8/47 (17.0%) (Figure 1B). Then, we further verified that the expression of SVEP1 in the tumor tissues was substantially lower than that in the adjacent normal tissues by using western blot assay in five paired ICC samples (Figure 1C).

Subsequently, the clinical analysis indicated that the decreased expression of SVEP1 in tumor tissues was an independent risk factor for the prognosis of ICC (DFS, *p* = .017; OS, *p* = .040) (Table 1). The estimated 5 year OS rates and DFS rates in the groups of the ICC patients with high and low expressions of SVEP1 were statistically significant using the Kaplan-Meier method. ICC patients with low-SVEP1 expression had a median OS of 34.7 months, which was much shorter than those with high-SVEP1 expression (*p* = .045) (Figure 1D). The difference in the DFS between the two groups is remarkable. The median DFS of the ICC patients with high SVEP1 expression was 38.4 months longer than that of the patients with low SVEP1 expression (*p* = .04) (Figure 1E). Thus, our data suggested that the low expression of SVEP1 might serve as a promising indicator in predicting high recurrence and low survival rate in ICC.

**TABLE 1:** Univariate and multivariate analysis of prognostic factors associated with OS and DFS in 47 ICC patients.

ICC patients (n = 47)	OS Univariate Analysis			OS Multivariate Analysis			DFS Multivariate Analysis			DFS Univariate Analysis		
	HR (log rank)	95% CI	*p*-value	HR (log rank)	95% CI	*p*-value	HR (log rank)	95% CI	*p*-value	HR (log rank)	95% CI	*p*-value
Age(years) ≥55/<55	1.059	0.444–2.528	0.897				1.214	0.632–2.333	0.559			
Sex male/female	0.807	0.333–1.959	0.636				0.620	0.322–1.195	0.149			
CA199(U/ml) ≥27 < 27	1.155	0.460–2.903	0.759				1.616	0.811–3.219	0.168			
CA242(IU/ml) ≥20/<20	0.812	0.320–2.061	0.661				1.110	0.572–2.155	0.756			
CEA(μg/ml)≥5/<5	2.011	0.826–4.897	0.124				1.493	0.743–3.000	0.257			
TBIL(μmol/ml) ≥21/<21	1.968	0.829–4.676	0.125				1.702	0.892–3.250	0.102			
DBIL(μmol/ml) ≥8/<8	1.048	0.344–3.196	0.934				1.370	0.599–3.133	0.453			
Tumor size(cm) > 5/≤5	0.516	0.165–1.616	0.249				1.644	0.796–3.396	0.174			
Tumor number>1/≤1	0.419	0.097–1.817	0.231				1.256	0.520–3.035	0.611			
Tumor thrombus Y/N	5.661	2.268–14.128	<0.001*	6.762	2.560–17.861	<0.001*	3.359	1.619–6.967	0.001*	3.474	1.631–7.398	0.001*
Lymph nodes metastasis Y/N	4.703	1.780–12.426	0.001*	6.001	2.049–17.575	0.001*	2.230	1.047–4.750	0.033*	1.793	0.833–3.858	0.135
Satellite nodule Y/N	0.674	0.155–2.919	0.595				1.451	0.558–3.776	0.442			
TNM stage IandII/IIIandIV	1.476	0.525–4.148	0.457				1.047	0.505–2.171	0.902			
Staining score of SVEP1≥6/<6	0.165	0.022–1.230	0.045*	0.074	0.009–0.626	0.017*	0.412	0.171–0.989	0.040*	0.395	0.163–0.958	0.040*

### 3.2 Decreased expression of SVEP1 is significantly correlated with abnormal neovascularization

**FIGURE 2:**
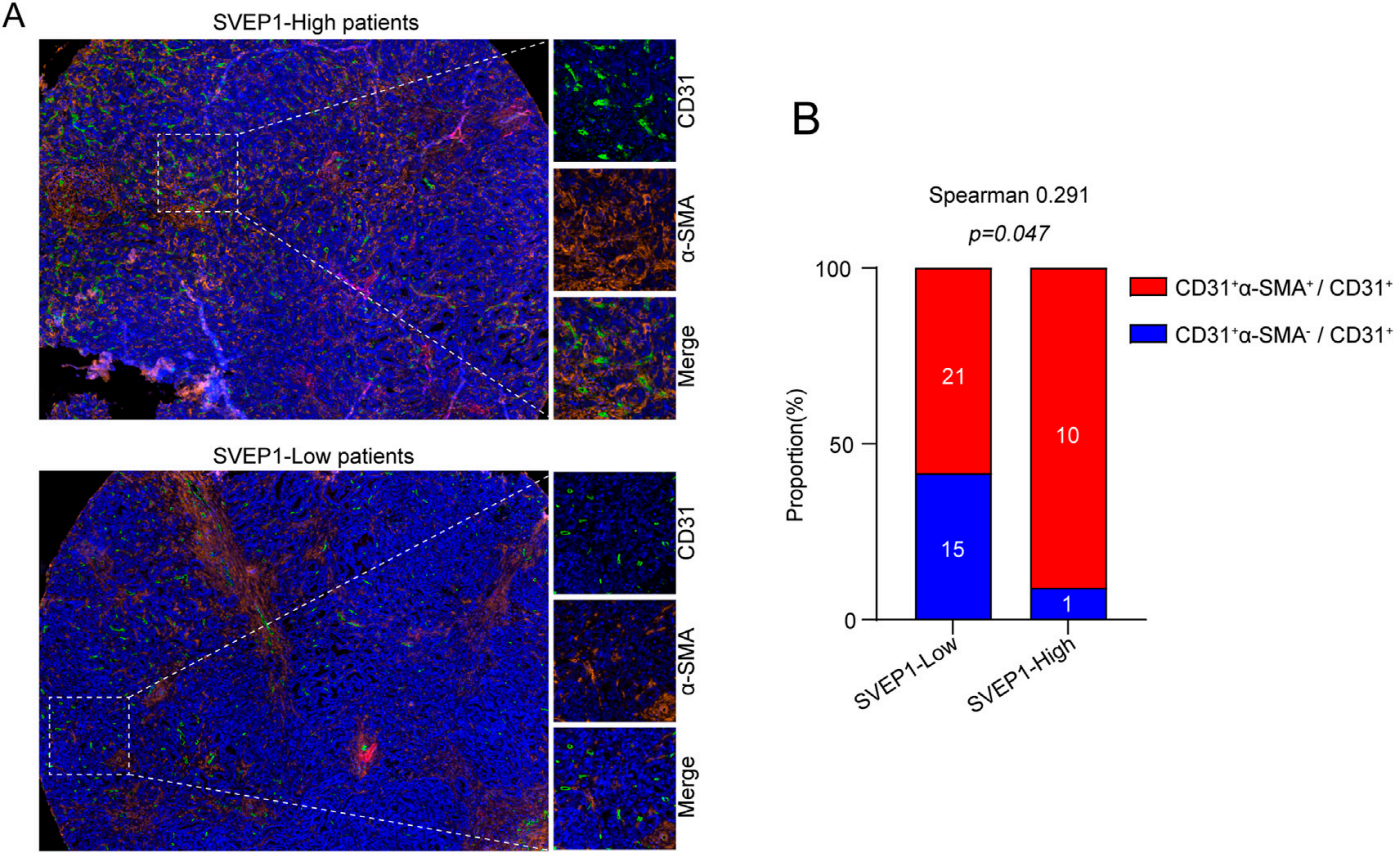
SVEP1 is correlated with vessel normalization. **(A)** Representative immunofluorescence images of CD31 (green), α-SMA (orange), and DAPI (blue) staining in tumor tissues with high expression of SVEP1 or low expression of SVEP1. **(B)** Immunofluorescent image quantification results. Relative proportions of α-SMA^+^ covered blood vessels in tumor tissues with high expression of SVEP1 or low expression of SVEP1, and the correlation analysis between CD31^+^α-SMA^+^ vessel rates and SVEP1 expression.

Generally, angiogenesis and structural abnormalities are key steps in the occurrence and metastasis of various malignant tumors, such as liver cancer and renal cancer (Cassetta and Pollard, 2018; Gong et al., 2022b). As an ECM molecule with protein interaction and adhesion functions, previous studies have identified zebrafish and mouse Polydom/SVEP1 as essential extracellular factors for lymphangiogenesis (Karpanen et al., 2017). However, their regulatory role in angiogenesis is still largely unknown and should be further explored. Here, we explored the relationship between SVEP1 expression and abnormal neovascularization in patients with ICC, and the results are exhibited in Figure 2. In general, the vascular smooth muscle cells supporting vascular endothelium (CD31^+^ and α-smooth muscle actin, α-SMA) play a key role in vascular maturation and maintenance of stability (Sun et al., 2021). The low expression of SVEP1 was dramatically correlated with increased proportion of CD31^+^ α-SMA-marked abnormal blood vessels in ICC (Figures 2A, B). The proportion of vascular smooth muscle cell covering the blood vessels in the SVEP1 low expression group was 58.3%, significantly lower than that in the SVEP1 high expression group (90.9%, *p* = .047, Figure 2B). In summary, these results suggested that decreased expression of SVEP1 might be an important link of intra-tumor abnormal neovascularization in ICC.

### 3.3 SVEP1 plays an important role in the maintenance of vascular stability likely though up-regulating the expression of Ang-1 in the ICC

**FIGURE 3:**
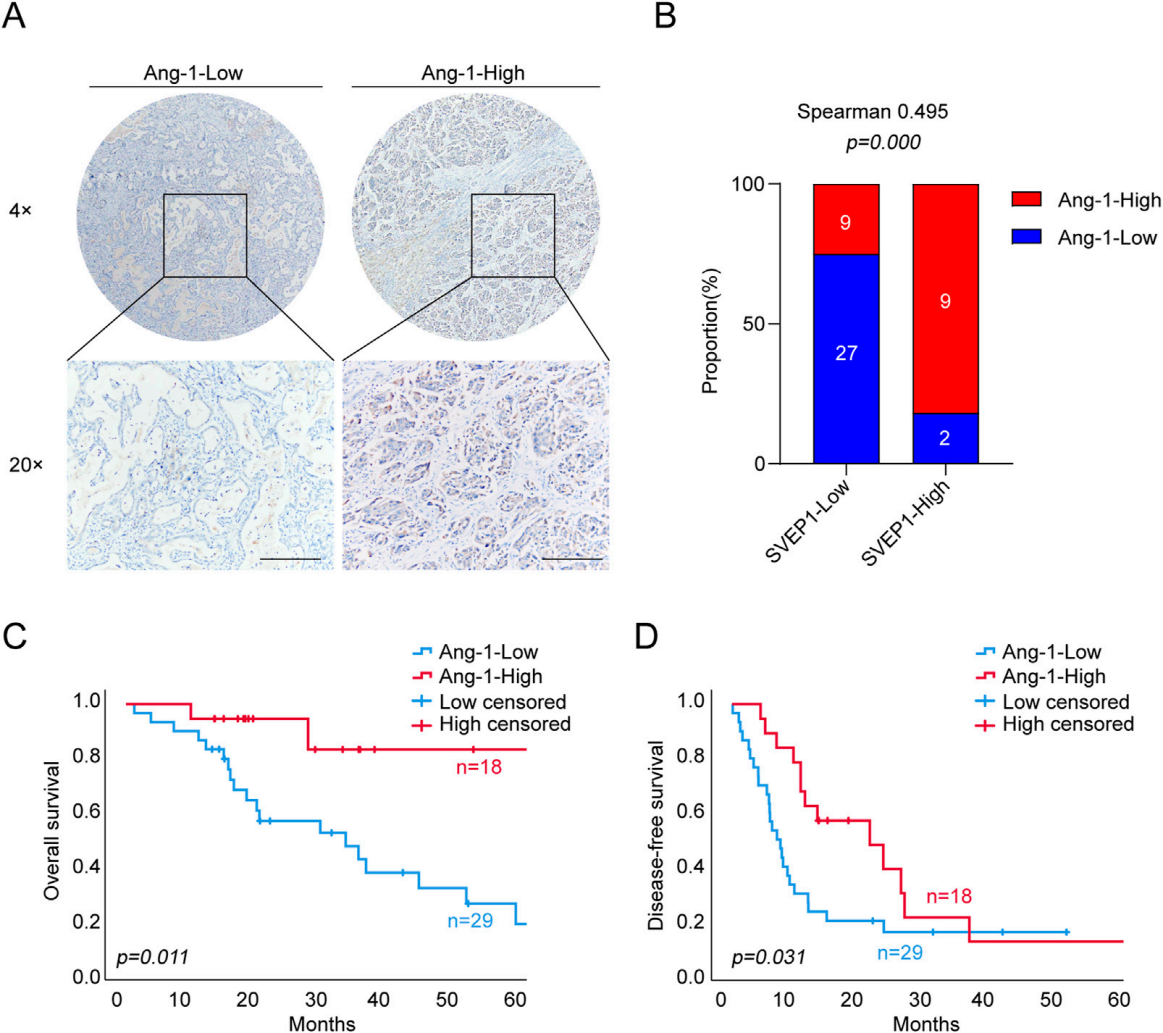
SVEP1 expression is positively related to the expression of Ang-1, which plays an important role in the maintenance of vascular stability. **(A)** Representative IHC images of Ang-1 in ICC tissues. **(B)** IHC image quantification results. Relative proportions of high and low levels of Ang-1 in the group of tumor tissues with high and low expression of SVEP1, and the correlation analysis between Ang-1 and SVEP1. **(C–D)** Correlation of Ang-1 expression with the OS **(C)** and DFS **(D)** time of ICC patients, respectively.

The angiopoietin-1/tyrosine kinase with immunoglobulin and epidermal growth factor homology domain (Ang-1/Tie) pathway is well known for its important role in stabilizing newly formed blood vessels and forming an efficient and stable vascular network (Joussen et al., 2021). We performed IHC staining to determine the expression of Ang-1 in all 47 ICC clinical samples to further explore the potential mechanism that SVEP1 might participate in the maintenance of vascular stability. The data are shown in Figure 3. The expression of SVEP1 in the ICC was dramatically correlated with Ang-1 expression (*p* = .000, Figures 3A, B). Nine out of 11 cases (81.8%) in the SVEP1 high expression group exhibited high Ang-1 expression, whereas for the SVEP1 low expression group, the proportion of Ang-1 high expression dropped to 25% (Figure 3B). Notably, the low expression of Ang-1 is also a risk factor for poor prognosis of ICC. Patients with decreased Ang-1 expression always had a shorter OS (*p* = .011) (Figure 3C) as well as DFS (*p* = .031) (Figure 3D). We hypothesized that SVEP1 might maintain vascular stability likely through up-regulating the expression of Ang-1 in the ICC because the high expression of SVEP1 in the ICC is almost accompanied by the high expression of Ang-1 and is significantly positively related to the prognosis of ICC patients.

### 3.4 Relationship between SVEP1 expression and malignant proliferation phenotype of the ICC

**FIGURE 4:**
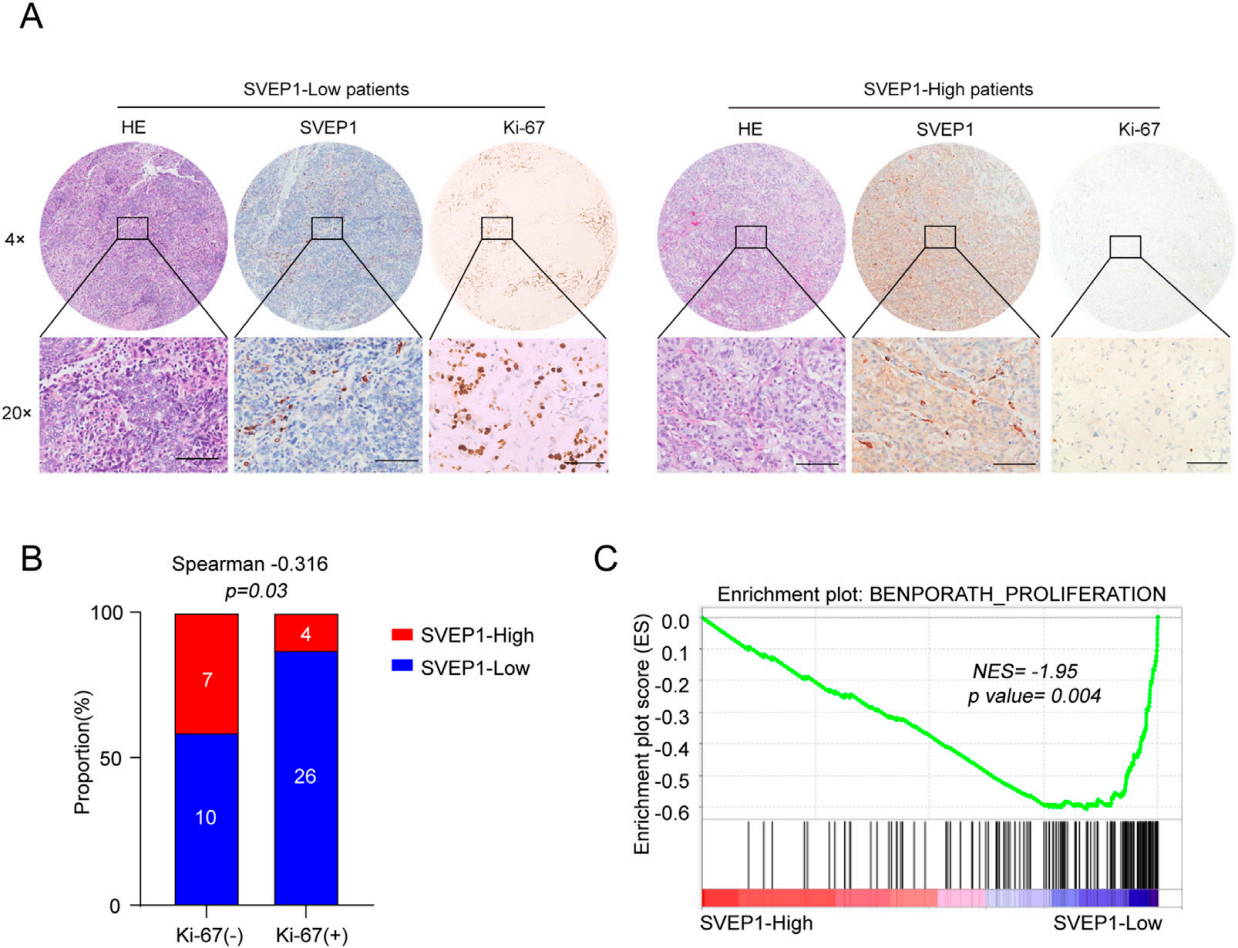
ICC samples with low expression of SVEP1 present malignant proliferation phenotype. **(A)** Representative HE and IHC images of SVEP1 and Ki-67 in ICC tissues. **(B)** Correlation analysis of Ki-67 expression with the SVEP1 level in ICC samples. **(C)** GSEA of correlation between the expression of SVEP1 and tumor proliferation.

Our previous studies demonstrated that the low-SVEP1 expression has an increased likelihood of having increased tumor sizes in patients with hepatocellular carcinoma (Chen et al., 2020). Consistently, in this study, we found that the low expression of SVEP1 was accompanied by an increase in Ki-67 expression in the ICC (Figure 4A; Table 2). Further studies have revealed that 26/30 (86.7%) cases in the Ki-67 high expression group exhibited low SVEP1 expression. However, in the Ki-67 low expression group, only 10/17 (58.8%) cases displayed low SVEP1 expression. The difference between the two groups was statistically significant (*p* = .03, Figure 4B). Then, a gene set enrichment analysis (GSEA) based on the mRNA data of the ICC samples from GSE132305 was performed to validate the correlation of the SVEP1 mRNA level and tumor cell proliferation phenotype. Figure 4C shows that the low expression of SVEP1 in the ICC was positively correlated with the high proliferation phenotype of tumor (*p* = .004; Figure 4C). These results suggested that the decreased expression of SVEP1 in the ICC patients indicated that tumors are likely to be in the malignant proliferation phenotype. This finding corroborates our previous clinical data, indicating that the low-SVEP1 expression patients have shorter OS and DFS from another aspect.

**TABLE 2:** Relationship between clinicopathological characteristics and SVEP1 expression in 47 ICC patients.

Characteristics		Total	SVEP1 expression			*p*-value	Characteristics		Total	SVEP1 expression		*p*-value
			Low		High					Low	High	
		47							47			
Tumor thrombus						0.494	Tumor size(cm)					0.269
	Present	17	14		3			>5	10	9	1	
	Absent	30	22		8			≤5	37	27	10	
CA199(U/ml)						0.988	Tumor number					0.733
	≥27	30	23		7			>1	7	5	2	
	<27	17	13		4			≤1	40	31	9	
CA242(IU/ml)						0.494	Satellite nodule					0.154
	≥20	17	14		3			Present	6	6	0	
	<20	30	22		8			Absent	41	30	11	
CEA(μg/ml)						0.839	Lymph nodes metastasis					0.343
	≥5	14	11		3			Present	9	8	1	
	<5	33	25		8			Absent	38	28	10	
TNM stage						0.649	Staining score of Ki-67					0.030*
	IandII	11	9		2			≥15%	30	26	4	
	IIIandIV	36	27		9			<15%	17	10	7	

## 4 Discussion

ICC is one of the most common malignancies with difficulty in early diagnosis and extremely poor prognosis (Zhou et al., 2019). Globally, Southeast Asia has the highest incidence of ICC, and the incidence rate is approximately 10 times that of Europe (Banales et al., 2016). The low incidence rate and high heterogeneity of ICC restrict the development of large-scale randomized prospective clinical research. In addition to surgical resection, gemcitabine/platinum is currently the standard first-line treatment for advanced cholangiocarcinoma, but the proportion of clinical benefits is limited, and the median survival of patients is less than 1 year (Valle et al., 2017). In recent years, although gemcitabine and cisplatin plus immunotherapy showed promising efficacy and acceptable safety in patients with ICC (Oh et al., 2022), the prognosis of some ICC remains poor. Novel diagnostic and prognostic markers for ICC with high malignancy and therapy resistance should be further identified.

SVEP1 protein is composed of 3571 amino acids and has multiple domains, including epidermal growth factor domain, transparent repeat domain, and pentameric protein domain (Song et al., 2016). Previous studies have shown that SVEP1 is a key molecule that mediates cell adhesion (Shefer and Benayahu, 2010). The knockout of SVEP1 in mice directly leads to the death of the embryos it incubates. Moreover, decreasing the expression of SVEP1 in keratinocytes will correspondingly reduce the expression of its epithelial marker molecules and affect the epithelial phenotype differentiation of cells (Samuelov et al., 2017). However, the studies of SVEP1 in tumorigenesis and development are very limited currently. The related role of SVEP1 in cancers was first reported in 2007. The research used chromatin immunoprecipitation (ChIP) to analyze the binding relationship between estrogen receptor (ER) and SVEP1 promoter and proposed that estrogen treatment would affect the binding of ER and SVEP1 promoter, thereby regulating the mRNA and protein expression level of SVEP1 in breast cancer cells (Shur et al., 2007). Then, further studies confirmed that 17βE2 and TNFα could promote the expression of SVEP1 by activating the promoter of SVEP1 in breast cancer cells (Glait-Santar and Benayahu, 2012). In addition, SVEP1 has been proven to play an important role in preventing tumor cells from invading bone niches as a key cell adhesion molecule (Glait-Santar et al., 2012). In our previous studies, we found and reported that SVEP1, as a promising novel diagnostic and prognostic biomarker, plays various regulatory roles in the malignant progression of hepatocellular carcinoma (Chen et al., 2020; Gong et al., 2022a; Guo et al., 2022). In this study, we verified that the expression level of SVEP1 was dramatically decreased in the ICC tumor tissues when compared with the corresponding adjacent tissues and that it was negatively correlated with OS and DFS by using IHC in a 47 ICC patient tissue microarray.

In the previous decades, most of the tumor research focused only on the tumor cells and suggested that the oncogene or tumor suppressor gene mutations that accumulated in the process of tumor evolution were the main reasons for tumor occurrence and progression. In recent years, more research results show that, in addition to the malignant biological characteristics of tumor cells, tumor microenvironment (TME) plays an important role in the process of tumor evolution (Hinshaw and Shevde, 2019). Among them, abnormal neovascularization in TME is an important feature of malignant tumors and is also the key factor leading to various treatment failures (Lamplugh and Fan, 2021). In fact, as early as 2008, Hamzah et al. pointed out that inhibiting the expression of G protein signal regulator 5 (Rgs5) can promote the normalization of tumor blood vessels, thereby significantly reducing tumor hypoxia and vascular leakage, facilitating the flow of immune effector cells into the tumor substance, and significantly prolonging the survival time of tumor-bearing mice (Hamzah et al., 2008). Similar research published in 2021 reported that Delta like 1 (DLL1) could promote the responsiveness of breast cancer and lung cancer to immunotherapy by mediating the normalization of tumor blood vessels and the polarization of M1-like macrophages (Zhang et al., 2021). SVEP1 has been proven to be necessary for lymphatic remodeling in previous studies (Morooka et al., 2017), but its role in angiogenesis has not been discussed previously. The data shown in this study suggested that decreased expression of SVEP1 was significantly correlated with abnormal neovascularization in the ICC. The proportion of normal blood vessels covered by smooth muscle cells (CD31^+^ α-SMA^+^) in the ICC samples in the low-SVEP1 expression group was significantly reduced when compared with that in the high-SVEP1 expression group (*p* = .047). Consistent with this finding, the SVEP1 deposited around the blood vessels seems to ensure Ang-1 upregulation in TME. The proportion of ICC patients with high Ang-1 expression in the SVEP1-high expression group was significantly higher than that in the SVEP1-low expression group (81.8% vs. 25%, *p* = .000). Generally, the Ang-1/Tie pathway is one of the most important pathways necessary for lymphatic and blood vessel development, and this pathway controls vascular permeability, inflammation, and pathological angiogenic responses in adult tissues (Saharinen et al., 2017). Our results showed that the ICC patients with low Ang-1 expression have shorter OS (*p* = .011) and DFS (*p* = .031) than that of the high Ang-1 expression group. In addition, ICC patients with low expression of SVEP1 also showed the characteristic of high proliferation in our study. All these findings suggested that decreased expression of SVEP1 is highly correlated with abnormal neovascularization and tumor cell high proliferation activity in the ICC and might provide a basis for the exploration of the mechanism of the poor prognosis and treatment resistance of ICC in further studies.

Nevertheless, this study still has several limitations. First, the number of cases is relatively small. Thus, we hope to include more clinical samples for further verification in our subsequent studies. Second, further molecular biological experiments are required to explore the underlying mechanism of abnormal neovascularization induced by the decreased expression of SVEP1 in ICC. However, the defects cannot obscure the virtues. In this study, we determined that SVEP1 was down-regulated in the ICC for the first time and verified that the low SVEP1 expression was related to abnormal neovascularization and poor prognosis. The relevant results of this study revealed the important value of SVEP1 for predicting prognosis and its promising application in normalizing blood vessels in ICC treatment.

## Data Availability

The original contributions presented in the study are included in the article/supplementary material, further inquiries can be directed to the corresponding authors.

## References

[B1] Banales J. M. Cardinale V. Carpino G. Marzioni M. Andersen J. B. Invernizzi P. (2016). Expert consensus document: Cholangiocarcinoma: Current knowledge and future perspectives consensus statement from the European network for the study of cholangiocarcinoma (ENS-CCA). Nat. Rev. Gastroenterol. Hepatol. 13 (5), 261–280. 10.1038/nrgastro.2016.51 27095655

[B2] Banales J. M. Marin J. J. G. Lamarca A. Rodrigues P. M. Khan S. A. Roberts L. R. (2020). Cholangiocarcinoma 2020: The next horizon in mechanisms and management. Nat. Rev. Gastroenterol. Hepatol. 17 (9), 557–588. 10.1038/s41575-020-0310-z 32606456 PMC7447603

[B3] Bejarano L. Jordāo M. J. C. Joyce J. A. (2021). Therapeutic targeting of the tumor microenvironment. Cancer Discov. 11 (4), 933–959. 10.1158/2159-8290.CD-20-1808 33811125

[B4] Cassetta L. Pollard J. W. (2018). Targeting macrophages: Therapeutic approaches in cancer. Nat. Rev. Drug Discov. 17 (12), 887–904. 10.1038/nrd.2018.169 30361552

[B5] Chen L. Fu H. Luo Y. Chen L. Cheng R. Zhang N. (2017). cPLA2α mediates TGF-β-induced epithelial-mesenchymal transition in breast cancer through PI3k/Akt signaling. Cell Death Dis. 8 (4), e2728. 10.1038/cddis.2017.152 28383549 PMC5477578

[B6] Chen L. Liu D. Yi X. Qi L. Tian X. Sun B. (2020). The novel miR-1269b-regulated protein SVEP1 induces hepatocellular carcinoma proliferation and metastasis likely through the PI3K/Akt pathway. Cell Death Dis. 11 (5), 320. 10.1038/s41419-020-2535-8 32371982 PMC7200779

[B7] Chen L. Tian X. Gong W. Sun B. Li G. Liu D. (2019). Periostin mediates epithelial-mesenchymal transition through the MAPK/ERK pathway in hepatoblastoma. Cancer Biol. Med. 16 (1), 89–100. 10.20892/j.issn.2095-3941.2018.0077 31119049 PMC6528457

[B8] Glait-Santar C. Benayahu D. (2012). Regulation of SVEP1 gene expression by 17β-estradiol and TNFα in pre-osteoblastic and mammary adenocarcinoma cells. J. Steroid Biochem. Mol. Biol. 130 (1-2), 36–44. 10.1016/j.jsbmb.2011.12.015 22265959

[B9] Glait-Santar C. Pasmanik-Chor M. Benayahu D. (2012). Expression pattern of SVEP1 alternatively-spliced forms. Gene 505 (1), 137–145. 10.1016/j.gene.2012.05.015 22659106

[B10] Gong W. Han Z. Fang F. Chen L. (2022). Yap expression is closely related to tumor angiogenesis and poor prognosis in hepatoblastoma. Fetal Pediatr. Pathol. 41 (6), 929–939. 10.1080/15513815.2021.2020384 34978260

[B11] Gong W-C. Han Z-Q. Guo M-X. Zhao S. Guo Y-H. Meng B. (2022). Decreased expression of SVEP1 is closely related to a cancer stem cell-like phenotype and poor prognosis in hepatocellular carcinoma. Neoplasma 69 (5), 1209–1216. 10.4149/neo_2022_220614N629 35900319

[B12] Guo L. Yi X. Chen L. Zhang T. Guo H. Chen Z. (2022). Single-cell DNA sequencing reveals punctuated and gradual clonal evolution in hepatocellular carcinoma. Gastroenterology 162 (1), 238–252. 10.1053/j.gastro.2021.08.052 34481846

[B13] Hamzah J. Jugold M. Kiessling F. Rigby P. Manzur M. Marti H. H. (2008). Vascular normalization in Rgs5-deficient tumours promotes immune destruction. Nature 453 (7193), 410–414. 10.1038/nature06868 18418378

[B14] He Y. Xiao M. Fu H. Chen L. Qi L. Liu D. (2020). cPLA2α reversibly regulates different subsets of cancer stem cells transformation in cervical cancer. Stem Cells 38 (4), 487–503. 10.1002/stem.3157 32100928

[B15] Hinshaw D. C. Shevde L. A. (2019). The tumor microenvironment innately modulates cancer progression. Cancer Res. 79 (18), 4557–4566. 10.1158/0008-5472.CAN-18-3962 31350295 PMC6744958

[B16] Joussen A. M. Ricci F. Paris L. P. Korn C. Quezada-Ruiz C. Zarbin M. (2021). Angiopoietin/Tie2 signalling and its role in retinal and choroidal vascular diseases: A review of preclinical data. Eye (Lond). 35 (5), 1305–1316. 10.1038/s41433-020-01377-x 33564135 PMC8182896

[B17] Kabir A. U. Subramanian M. Lee D. H. Wang X. Krchma K. Wu J. (2021). Dual role of endothelial *Myct1* in tumor angiogenesis and tumor immunity. Sci. Transl. Med. 13 (583), eabb6731. 10.1126/scitranslmed.abb6731 33658356 PMC8252962

[B18] Karpanen T. Padberg Y. van de Pavert S. A. Dierkes C. Morooka N. Peterson-Maduro J. (2017). An evolutionarily conserved role for polydom/svep1 during lymphatic vessel formation. Circ. Res. 120 (8), 1263–1275. 10.1161/CIRCRESAHA.116.308813 28179432 PMC5389596

[B19] Lamplugh Z. Fan Y. (2021). Vascular microenvironment, tumor immunity and immunotherapy. Front. Immunol. 12, 811485. 10.3389/fimmu.2021.811485 34987525 PMC8720970

[B20] Li S. Liu M. Do M. H. Chou C. Stamatiades E. G. Nixon B. G. (2020). Cancer immunotherapy via targeted TGF-β signalling blockade in TH cells. Nature 587 (7832), 121–125. 10.1038/s41586-020-2850-3 33087933 PMC8353603

[B21] Mohan V. Das A. Sagi I. (2020). Emerging roles of ECM remodeling processes in cancer. Semin. Cancer Biol. 62, 192–200. 10.1016/j.semcancer.2019.09.004 31518697

[B22] Morooka N. Futaki S. Sato-Nishiuchi R. Nishino M. Totani Y. Shimono C. (2017). Polydom is an extracellular matrix protein involved in lymphatic vessel remodeling. Circ. Res. 120 (8), 1276–1288. 10.1161/CIRCRESAHA.116.308825 28179430

[B23] Nakamura H. Arai Y. Totoki Y. Shirota T. Elzawahry A. Kato M. (2015). Genomic spectra of biliary tract cancer. Nat. Genet. 47 (9), 1003–1010. 10.1038/ng.3375 26258846

[B24] Oh D-Y. Lee K-H. Lee D-W. Yoon J. Kim T-Y. Bang J-H. (2022). Gemcitabine and cisplatin plus durvalumab with or without tremelimumab in chemotherapy-naive patients with advanced biliary tract cancer: An open-label, single-centre, phase 2 study. Lancet Gastroenterol. Hepatol. 7 (6), 522–532. 10.1016/S2468-1253(22)00043-7 35278356

[B25] Saharinen P. Eklund L. Alitalo K. (2017). Therapeutic targeting of the angiopoietin-TIE pathway. Nat. Rev. Drug Discov. 16 (9), 635–661. 10.1038/nrd.2016.278 28529319

[B26] Samuelov L. Li Q. Bochner R. Najor N. A. Albrecht L. Malchin N. (2017). SVEP1 plays a crucial role in epidermal differentiation. Exp. Dermatol 26 (5), 423–430. 10.1111/exd.13256 27892606 PMC5543306

[B27] Sasaki T. Takeda T. Okamoto T. Ozaka M. Sasahira N. (2021). Chemotherapy for biliary tract cancer in 2021. J. Clin. Med. 10 (14), 3108. 10.3390/jcm10143108 34300274 PMC8305063

[B28] Shefer G. Benayahu D. (2010). SVEP1 is a novel marker of activated pre-determined skeletal muscle satellite cells. Stem Cell Rev. Rep. 6 (1), 42–49. 10.1007/s12015-009-9106-9 20052625

[B29] Shur I. Zemer-Tov E. Socher R. Benayahu D. (2007). SVEP1 expression is regulated in estrogen-dependent manner. J. Cell Physiol. 210 (3), 732–739. 10.1002/jcp.20895 17139625

[B30] Song D. Cretoiu D. Zheng M. Qian M. Zhang M. Cretoiu S. M. (2016). Comparison of Chromosome 4 gene expression profile between lung telocytes and other local cell types. J. Cell Mol. Med. 20 (1), 71–80. 10.1111/jcmm.12746 26678350 PMC4717865

[B31] Sun Y. Chen W. Torphy R. J. Yao S. Zhu G. Lin R. (2021). Blockade of the CD93 pathway normalizes tumor vasculature to facilitate drug delivery and immunotherapy. Sci. Transl. Med. 13 (604), eabc8922. 10.1126/scitranslmed.abc8922 34321321 PMC8749958

[B32] Sung H. Ferlay J. Siegel R. L. Laversanne M. Soerjomataram I. Jemal A. (2021). Global cancer statistics 2020: GLOBOCAN estimates of incidence and mortality worldwide for 36 cancers in 185 countries. CA Cancer J. Clin. 71 (3), 209–249. 10.3322/caac.21660 33538338

[B33] Sung Y-C. Jin P-R. Chu L-A. Hsu F-F. Wang M-R. Chang C-C. (2019). Delivery of nitric oxide with a nanocarrier promotes tumour vessel normalization and potentiates anti-cancer therapies. Nat. Nanotechnol. 14 (12), 1160–1169. 10.1038/s41565-019-0570-3 31740794

[B34] Valle J. W. Lamarca A. Goyal L. Barriuso J. Zhu A. X. (2017). New horizons for precision medicine in biliary tract cancers. Cancer Discov. 7 (9), 943–962. 10.1158/2159-8290.CD-17-0245 28818953 PMC5586506

[B35] Zhang N. Yin R. Zhou P. Liu X. Fan P. Qian L. (2021). DLL1 orchestrates CD8^+^ T cells to induce long-term vascular normalization and tumor regression. Proc. Natl. Acad. Sci. U. S. A. 118 (22), e2020057118. 10.1073/pnas.2020057118 34035167 PMC8179177

[B36] Zhou G. Sprengers D. Mancham S. Erkens R. Boor P. P. C. van Beek A. A. (2019). Reduction of immunosuppressive tumor microenvironment in cholangiocarcinoma by *ex vivo* targeting immune checkpoint molecules. J. Hepatol. 71 (4), 753–762. 10.1016/j.jhep.2019.05.026 31195061

